# Fine Pathogen Discrimination within the APL1 Gene Family Protects *Anopheles gambiae* against Human and Rodent Malaria Species

**DOI:** 10.1371/journal.ppat.1000576

**Published:** 2009-09-11

**Authors:** Christian Mitri, Jean-Claude Jacques, Isabelle Thiery, Michelle M. Riehle, Jiannong Xu, Emmanuel Bischoff, Isabelle Morlais, Sandrine E. Nsango, Kenneth D. Vernick, Catherine Bourgouin

**Affiliations:** 1 Unit of Insect Vector Genetics and Genomics, Department of Parasitology and Mycology, CNRS Unit URA3012: Hosts, Vectors and Infectious Agents, Institut Pasteur, Paris, France; 2 Center for the Production and Infection of Anopheles, Institut Pasteur, Paris, France; 3 Department of Microbiology, University of Minnesota, Saint Paul, Minnesota, United States of America; 4 Laboratoire de Recherche sur le Paludisme, Institut de Recherche pour le Développement IRD-OCEAC, Yaoundé, Cameroun; Stanford University, United States of America

## Abstract

Genetically controlled resistance of *Anopheles gambiae* mosquitoes to *Plasmodium falciparum* is a common trait in the natural population, and a cluster of natural resistance loci were mapped to the *Plasmodium*-Resistance Island (PRI) of the *A. gambiae* genome. The APL1 family of leucine-rich repeat (LRR) proteins was highlighted by candidate gene studies in the PRI, and is comprised of paralogs *APL1A*, *APL1B* and *APL1C* that share ≥50% amino acid identity. Here, we present a functional analysis of the joint response of *APL1* family members during mosquito infection with human and rodent *Plasmodium* species. Only paralog *APL1A* protected *A. gambiae* against infection with the human malaria parasite *P. falciparum* from both the field population and *in vitro* culture. In contrast, only paralog *APL1C* protected against the rodent malaria parasites *P. berghei* and *P. yoelii*. We show that anti-*P. falciparum* protection is mediated by the Imd/Rel2 pathway, while protection against *P. berghei* infection was shown to require Toll/Rel1 signaling. Further, only the short Rel2-S isoform and not the long Rel2-F isoform of Rel2 confers protection against *P. falciparum*. Protection correlates with the transcriptional regulation of *APL1A* by Rel2-S but not Rel2-F, suggesting that the Rel2-S anti-parasite phenotype results at least in part from its transcriptional control over *APL1A*. These results indicate that distinct members of the *APL1* gene family display a mutually exclusive protective effect against different classes of *Plasmodium* parasites. It appears that a gene-for-pathogen-class system orients the appropriate host defenses against distinct categories of similar pathogens. It is known that insect innate immune pathways can distinguish between grossly different microbes such as Gram-positive bacteria, Gram-negative bacteria, or fungi, but the function of the APL1 paralogs reveals that mosquito innate immunity possesses a more fine-grained capacity to distinguish between classes of closely related eukaryotic pathogens than has been previously recognized.

## Author Summary

The African malaria vector mosquito *Anopheles gambiae* possesses immune mechanisms that can protect it against infection with malaria parasites, which kill more than one million people per year. Much work studying mosquito response to malaria has used model rodent malaria parasites that do not infect people, but are in the same genus and overall share most major features with the human parasites. Here, we show that the immune response used by *A. gambiae* to protect itself against infection by human and rodent malaria parasites utilizes different immune signaling pathways. A family of proteins called APL1 appears to be responsible for the ability of the mosquito to distinguish between the human or rodent malaria parasites. An individual APL1 relative is required for protection against the human malaria parasite but has no effect against the rodent parasite, and another APL1 relative is required for protection against rodent but not human malaria. This represents the finest ability yet demonstrated of mosquito immunity to distinguish between relatively similar pathogens, and highlights the distinct nature of mosquito response against human as compared to rodent malaria parasites.

## Introduction

Malaria remains a major global health problems, with more than 300 million estimated cases and more than one million deaths annually [1]. *Plasmodium falciparum* is the most common human malaria parasite in Africa and is responsible for the majority of mortality due to malaria. The vector mosquito, *Anopheles gambiae*, is widespread in sub-Saharan Africa, where it constitutes the major vector of *P. falciparum*. Vector control has been one of the foundations of malaria control. However, recent problems such as the rapid spread of drug-resistant parasites and insecticide-resistant mosquitoes have encouraged the development of a new generation of vector-based malaria control tools. Such new vector-based strategies might be based on the introduction into the vector population of genetic information encoding novel desirable traits that reduce transmission [2], or on exploiting natural selection to promote the expansion of pre-existing desirable traits [3],[4].

The high level of *P. falciparum* transmission in Africa, which is sustained in large part by *A. gambiae*, led to the idea that this mosquito species is generally permissive for the development of *P. falciparum*. However, analysis of the field population indicates that genetically-controlled resistance of wild *A. gambiae* mosquitoes to *P. falciparum* in West Africa is relatively frequent [5]. Genetic mapping led to the identification of major-effect loci for *P. falciparum* resistance that cluster within the PRI of the *A. gambiae* genome [4]. This same genomic region was also identified in East African *A. gambiae* as a major locus controlling *P. falciparum* development [6],[7]. The identification of common genetic control over susceptibility to *P. falciparum* infection in geographically distant *A. gambiae* populations suggests that a shared and widespread resistance mechanism operates in *A. gambiae* throughout sub-Saharan Africa. Such a widespread trait might be an evolutionarily old host-defense mechanism that could have been shaped and maintained by selective pressure from malaria or other similar pathogens.

A candidate gene study of the ∼1000 predicted protein coding sequences in the ∼15 Mb PRI locus yielded two genes that survived all filter criteria [4]. These two genes, named *APL1* and *APL2*, encode LRR proteins, which is a widespread motif in innate immune proteins among animals and plants [8]. RNAi-mediated gene silencing in an *A. gambiae* laboratory strain indicated that candidate gene *APL1*, but not *APL2*, was highly protective against the development of the rodent malaria parasite, *P. berghei* [4]. Subsequent reannotation of the *APL1* locus revealed the original *APL1* “gene” to be three paralogous members of a gene family, which were then named *APL1A*, *APL1B* and *APL1C* [9]. Gene-specific knockdown assays showed that of the three paralogs, only *APL1C* was responsible for the previously observed control over *P. berghei* parasite intensity and infection prevalence.

Here, we functionally analyzed the joint response of *APL1* family members during mosquito infection with both human and rodent *Plasmodium* species. First, using a field assay system we discovered that the action of the *APL1* family conferred mosquito resistance against wild *P. falciparum*. We then transferred the same mosquito assay strain to the laboratory where we challenged mosquitoes with *in vitro* cultured *P. falciparum* gametocytes, and observed equivalent APL1 protection as against the wild parasites. This provided a field-to-lab validation trail for the biological fidelity of the assay system, and justified use of the system for detailed laboratory dissection of APL1-based protection. To date, a small number of genes ascertained by laboratory studies have been shown to protect *A. gambiae* against *P. falciparum* [10]–[12]. A unique aspect of the current work is that we instead ascertained candidate *P. falciparum* resistance genes by initially genetically screening the natural population, and then extracted the trait from field to lab for functional dissection.

We report that of the three *APL1* family members, only paralog *APL1A* displayed a host-protective effect against *P. falciparum*, which we show is mediated by a specific sub-circuit of the Rel2 signalling pathway. In contrast, we extend the observation that only *APL1C* was responsible for control of *P. berghei* infection, which is mediated by the Rel1 pathway, with another rodent malaria species, *P. yoelii*. Thus, distinct members of the APL1 family of LRR proteins display a mutually exclusive protective effect against different classes of *Plasmodium* parasites, in the context of distinct immune signalling pathways.

## Results

### *APL1* controls the development of natural *P. falciparum* isolates in *A. gambiae*

**Figure 1:**
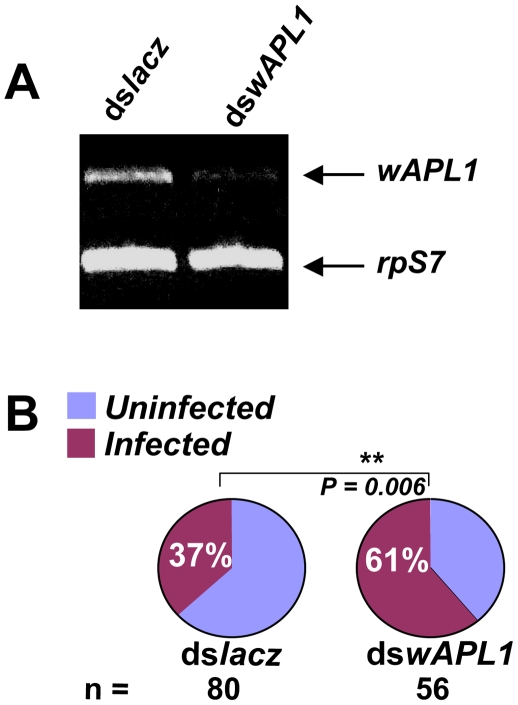
APL1 protects *A. gambiae* against wild *P. falciparum*. A) Expression of the APL1 gene family detected by RT-PCR using primers that amplify a conserved region shared by all three APL1 genes. Mosquitoes were injected with a dsRNA directed against the shared region to simultaneously silence the three APL1 paralogs (*dswAPL1*), or with an irrelevant control dsRNA (*dsLacz*). In this and subsequent figures, the injected dsRNA is indicated above the gel columns and the transcript detected by RT-PCR is indicated to the right of the relevant gel row. Decreased signal in row wAPL1 indicates that *dswAPL1* efficiently silences the entire APL1 family as compared to *dsLacZ*-injected controls. Detection of mRNA for ribosomal protein *S7* (rpS7) was used as an internal reference. B) Silencing of the *APL1* gene family permits significantly higher infection prevalence in mosquitoes fed on wild *P. falciparum* gametocytes from the natural population in Cameroon. There was no detectable effect of *dswAPL1* on parasite intensity. Infection prevalence effects are depicted by pie charts rather than histograms of parasite number because the significant difference is between infected or uninfected states, not parasite intensity (n, number of dissected mosquitoes; statistical descriptions of infections are presented in Supplementary Tables S2 and S3).

We tested the role of the *APL1* gene family in mosquito susceptibility to *P. falciparum* using wild parasites and a colonized strain of *A. gambiae*. A common *APL1* family domain is highly enough conserved at the nucleic acid level that a double-stranded RNA, *dswAPL1*, efficiently silences all three paralogs. We used *dswAPL1* to analyze the summed role of the *APL1* family on the development of wild *P. falciparum* in the recently colonized Ngousso strain of *A. gambiae*. Mosquitoes injected with *dswAPL1* were challenged by feeding on the blood of naturally-infected *P. falciparum* gametocyte carriers in Cameroon. The effect of *APL1* silencing on *P. falciparum* development was measured by determining oocyst number 7–8 d post-bloodmeal. Silencing of the entire *APL1* family resulted in a significant increase of infection prevalence (Figure 1; p = 0.006).

All gene silencing experiments presented herein were done as at least three biological replicates of at least 30 dissected mosquitoes per replicate (see Methods). Replicates were first analyzed independently to determine if there were significant differences between replicates within treatments: if not, data were pooled and analyzed statistically; if so, pooling is not statistically valid and replicates were instead analyzed independently and resulting p values were combined by meta-analysis (see Methods). The threshold of significance was defined conservatively as p = 0.01. Statistical details are summarized in Supplementary Tables S2 and S3. Infection prevalence is the fraction of mosquitoes carrying at least one oocyst, while parasite intensity is the number of oocysts per mosquito determined only in the subset of mosquitoes with ≥1 oocyst (see Methods). The *APL1* knockdown phenotype controlled only infection prevalence, with no detectable effect on oocyst intensity (p-value for intensity = 0.926). This result indicates that wild-type expression of the *APL1* gene family has a host-protective effect against natural genotypes of *P. falciparum* circulating in the field population.

### Paralog *APL1A* controls a developmental blockade of *P. falciparum*

Having found by knockdown of the entire gene family that *APL1* is protective against *P. falciparum*, we wished to determine the relative phenotypic contribution of the *APL1* paralogs. For this, we moved the experimental system to the laboratory to take advantage of the ease and reproducibility of *P. falciparum in vitro* gametocyte culture for infection of the same Ngousso mosquito strain used in Cameroon.

**Figure 2:**
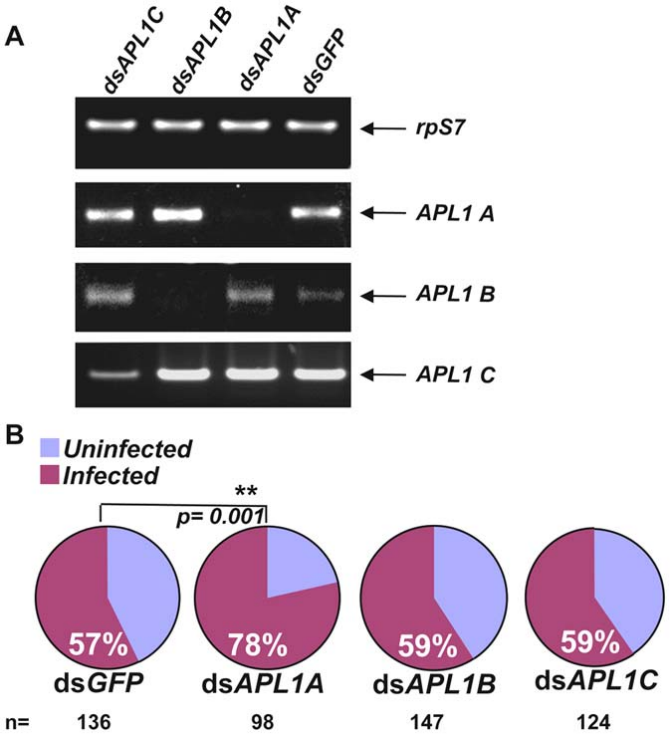
Paralog *APL1A* limits *P. falciparum* development in *A. gambiae*. A) Injection of dsRNAs that individually target *APL1A*, *APL1B*, or *APL1C* specifically diminish transcript levels from the cognate genes. B) Silencing of *APL1A* but not *APL1B* or *APL1C* permits significantly higher infection prevalence in mosquitoes fed on *in vitro* cultured *P. falciparum* gametocytes, with no detectable effect on parasite intensity.

There is sufficient nucleotide sequence divergence outside the *APL1* common domain to permit paralog-specific gene silencing [9]. Targeting non-conserved regions, we produced specific dsRNAs that diminish the expression of one paralog without affecting the expression of the other two (Figure 2A). Of the three genes, only the *APL1A* knockdown displayed a phenotype after *P. falciparum* challenge, with significantly higher prevalence of *P. falciparum* infection as compared to controls (Figure 2B; p = 0.001). Similar to the result described above for *dswAPL1* and wild *P. falciparum*, the phenotype of *dsAPL1A* controlled infection prevalence, not parasite intensity. Knockdowns of *APL1B* or *APL1C* produced phenotypes indistinguishable from the *dsGFP*-injected controls, indicating that the functions of these two paralogs have no effect on either *P. falciparum* prevalence of infection or parasite intensity.

**Figure 3:**
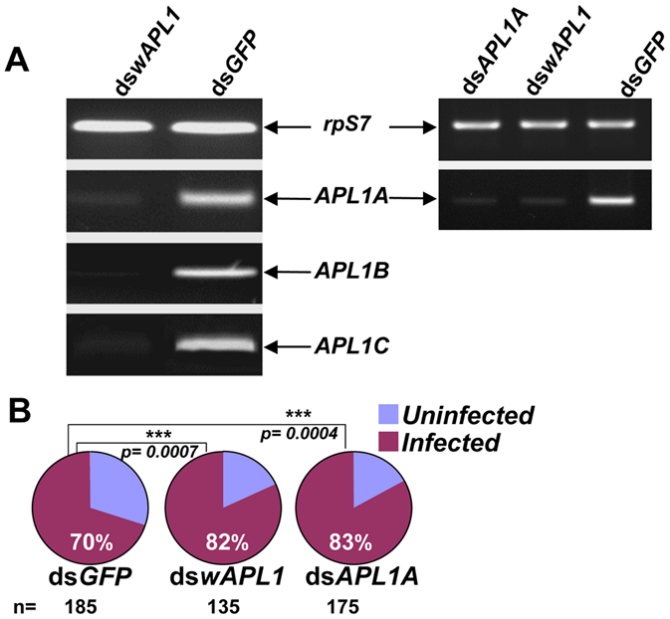
The effect of paralog *APL1A* alone is sufficient to explain APL1 family protective function against *P. falciparum*. A) RT-PCR of individual *APL1* paralogs confirms that *dswAPL1* efficiently silences all three *APL1* family members. Right panel verifies directly that *APL1A* is silenced with equivalent efficiency by either *dsAPL1A* or *dswAPL1*. B) Treatment of mosquitoes with either *dswAPL1* or *dsAPL1A* causes equivalent reduction in host protection against *P. falciparum* infection.

The above results suggest that the paralog *APL1A* is solely responsible for the host protective effect observed in the *dswAPL1* comprehensive knockdown of the *APL1* gene family. Because the two dsRNAs were tested in different experimental contexts (*dswAPL1* against wild parasites in Cameroon, *dsAPL1A* against gametocyte culture in the lab), we directly compared them by dsRNA treatment followed by challenge with cultured gametocytes in the same experimental infections. We verified that *dswAPL1* silences all three *APL1* paralogs, and that expression of *APL1A* was diminished with equivalent efficiency by treatment with either *dswAPL1* or *dsAPL1A* (Figure 3A). After infection, both *dswAPL1* and *dsAPL1A* produced equivalent, significantly higher *P. falciparum* infection prevalence than the *dsGFP* controls (Figure 3B; *dsAPL1A*, p = 0.0004; *dswAPL1*, p = 0.0007). This result confirms that of the *APL1* family, only *APL1A* acts as a host protective factor to limit infection with *P. falciparum* derived either from the field population or from *in vitro* gametocyte culture.

Interestingly, APL1A protection appears to operate with comparable efficiency against a multiplicity of parasite genotypes. The replicates of the *dswAPL1* treatment were each infected with blood from a different naturally-infected gametocyte carrier in Cameroon, who harbored different parasite genotypes (data not shown), and yet the combined replicates of the *dswAPL1* treatment displayed significantly elevated infection prevalence. Individually, each of the replicates also displayed the same trend of increased prevalence, although not significantly so, probably due to the reduced sample size. In addition, the *dsAPL1A*-treated mosquitoes were challenged with cultured gametocytes of the NF54 strain, which also contains multiple genotypes [13],[14]. Thus, the APL1A-mediated mechanism appears to protect mosquitoes against *P. falciparum* in a way that is probably general and not highly sensitive to parasite strain and genotype variation.

### *APL1C* protects against *P. berghei* and *P. yoelii* infection

**Figure 4:**
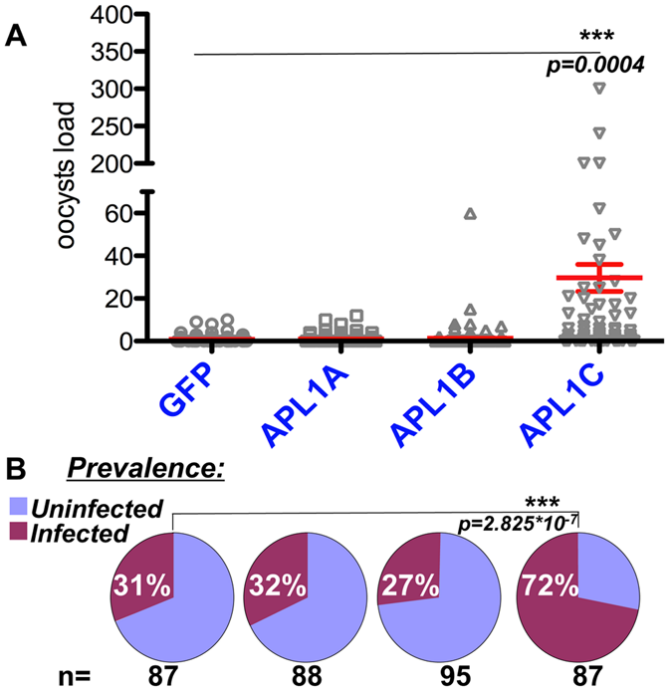
Paralog *APL1C* limits development of rodent malaria species in *A. gambiae*. A) Silencing of *APL1C* but not *APL1A* or *APL1B* permits significantly higher oocyst intensity in mosquitoes fed on mice infected with *P. berghei*. B) Silencing of *APL1C* also permits significantly higher *P. berghei* infection prevalence. Increases in both parasite intensity and infection prevalence were observed for another rodent malaria parasite, *P. yoelii*, following silencing of *APL1C* but not *APL1A* or *APL1B* (see Results).

We previously demonstrated that *APL1* family protection against the rodent malaria parasite *P. berghei* was mediated only by paralog *APL1C* in the G3 strain of *A. gambiae* [9]. In order to eliminate the formal possibility that the result obtained in G3 was a mosquito strain effect, we tested protection by the *APL1* paralogs against *P. berghei* in the Ngousso mosquito strain. In contrast to the results obtained above for *P. falciparum*, and identically to the results obtained in the G3 strain for *P. berghei*, the *APL1C* paralog also strongly limits infection with *P. berghei* in Ngousso mosquitoes (Figure 4; infection prevalence, p = 2.825×10^−7^; oocyst intensity, p = 0.0004). In addition, we obtained an equivalent result showing APL1C protection against *P. berghei* in a third strain of *A. gambiae*, the Yaoundé strain (data not shown). Thus, APL1C-based protection against *P. berghei* appears to be a general phenomenon, and is not restricted to a particular mosquito strain-parasite genetic combination as would be expected if APL1C protection displayed a large genotype-by-genotype effect.

We examined APL1 protection in Ngousso mosquitoes against another species of rodent malaria parasite, *P. yoelii*, which typically infects *A. gambiae* with a mixture of normal and melanized oocysts [15]. The *dsGFP*-treated controls permitted a median of 1 normal *P. yoelii* oocyst with 46% infection prevalence for normal oocysts and 48% harboring melanized parasites, while *dsAPL1C*-treated mosquitoes permitted a median of 28 normal oocysts with 86% infection prevalence for normal oocysts and 1.5% harboring melanized parasites, (*dsAPL1C* infection prevalence for normal oocysts, p = 6.2×10^−12^; intensity of normal oocysts, p = 1.7×10^−8^; presence of melanized parasites, p = 6.8×10^−7^). The *APL1A* or *APL1B* knockdowns were not significantly different from *dsGFP* controls for normal oocyst infection prevalence or intensity. Thus, APL1C is required for control of infection prevalence and oocyst intensity against *P. yoelii* similar to *P. berghei*, but with the difference that at least part of the APL1C-dependent protection against *P. yoelii* is mediated by the melanization response, which in this case is also APL1C-dependent. Overall, these results indicate that among the *APL1* paralogs, *APL1A* is exclusively responsible for establishing the permissive or non-permissive state for *P. falciparum* infection, with no detectable effect on *P. berghei* or *P. yoelii* infection; while *APL1C* among the paralogs limits infection by *P. berghei* and *P. yoelii*, but has no detectable influence upon *P. falciparum* development.

### Rel2 controls *P. falciparum* infection prevalence

In *Drosophila*, the Toll and Imd immune pathways are responsible for protection against a broad range of pathogens, including fungi, Gram-positive and Gram-negative bacteria [16]. When these pathways are activated, immune signals traverse multiple intracellular intermediates and are ultimately converted into regulated gene expression by the action of transcription factors Dif and Relish, respectively. Orthologs and homologs of some of these *Drosophila* factors have been identified in *A. gambiae* [17],[18]. At the terminus of the *A. gambiae* Toll pathway, Rel1 (also known as Gambif1) serves as the functional analogue of *Drosophila* Dif (no direct Dif ortholog has been identified in *A. gambiae*), while Rel2 is orthologous to the *Drosophila* transcription factor Relish [19]–[21].

**Figure 5:**
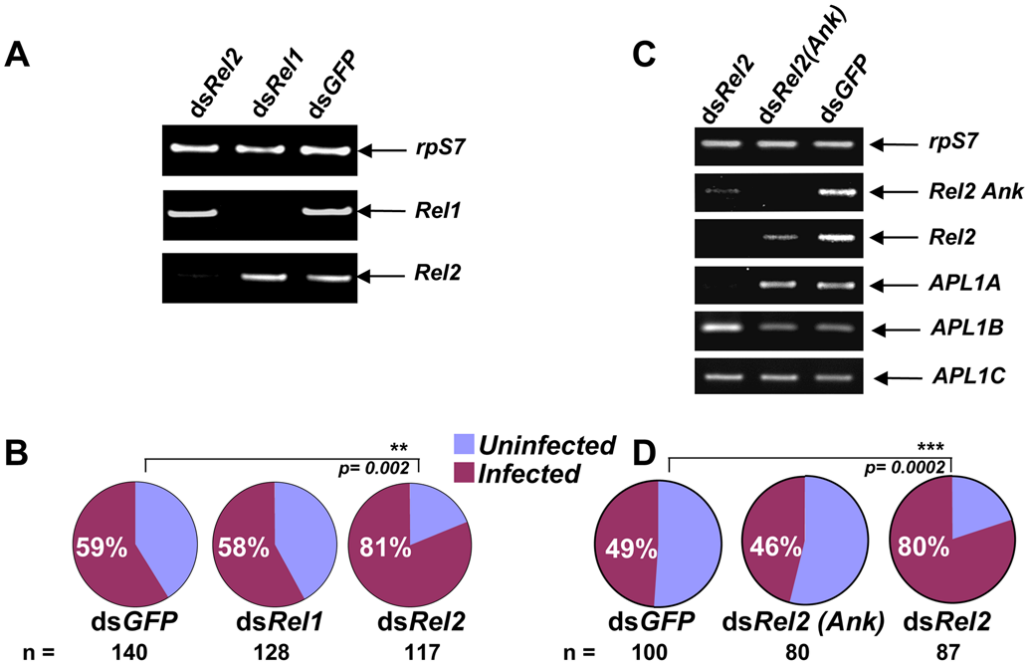
The major *A. gambiae* defense against *P. falciparum* is controlled by the short isoform of the immune signalling factor Rel2, which regulates *APL1A* expression. A) Gene silencing of immune signalling factors Rel1 and Rel2 is specific for the targeted genes. B) Silencing of *Rel2* but not *Rel1* permits significantly higher *P. falciparum* infection prevalence in treated mosquitoes. C) Genetic interactions between *Rel2* isoforms and *APL1* paralogs. *dsRel2* targets a common region shared by *Rel2-Short* and the ankyrin-domain containing *Rel2-Full* transcript isoforms. *dsRel2* treatment diminishes both of the *Rel2* transcripts, and also specifically silences *APL1A* but not *APL1B* or *APL1C* (RT-PCR in the horizontal row labelled Rel2 detects both *Rel2* transcripts, while the row labelled Rel2(Ank) detects only the longer *Rel2-Full* form). In contrast, treatment with *dsRel2(Ank)* efficiently diminishes the cognate long-form transcript (RT-PCR in row labelled Rel2(Ank)) and presumably the long-form proportion of the total *Rel2* transcripts detected by Rel2 RT-PCR (row Rel2), but does not affect transcript levels of *APL1A*, *APL1B*, or *APL1C*. D) Of the *Rel2* isoforms, only the short-form *Rel2-S* is required both for *APL1A* expression and for protection against *P. falciparum* infection. *dsRel2* treatment causes significantly higher infection prevalence, with no detectable effect on oocyst intensity. *dsRel2(Ank)* targets only the long-form transcript, and does not affect *APL1A* levels nor influence parasite infection.

Protection of *A. gambiae* against infection by *P. berghei* is largely mediated by Rel1 [9],[20], which translocates to the nucleus and activates transcription after the degradation of its cytoplasmic inhibitor, Cactus [22]. We previously showed that *APL1C* expression is required for Rel1-mediated protection against *P. berghei* [9]. Here, we first asked whether Rel1 or Rel2-mediated immune signalling controls the development of *P. falciparum* in *A. gambiae*. We silenced the ultimate transcription factor of each pathway in the Ngousso mosquito strain, followed by challenge with cultured *P. falciparum* gametocytes. Only knockdown of *Rel2*, and not *Rel1*, produced a phenotype, leading to a significant increase in infection prevalence compared to *dsGFP*-injected controls (Figure 5A&B, Rel2 p = 0.0002, Rel1 p = 0.908). Next, we constitutively activated Rel1 by silencing the gene for its inhibitor Cactus, followed by *P. falciparum* infection. The *dsCactus*-treated mosquitoes, with constitutively activated Rel1, displayed no *P. falciparum* infection difference as compared to controls (Supplementary Figure S1). Together, these results indicate that the Rel2 pathway, and not Rel1, is responsible for anti-*P. falciparum* protection, which is the inverse of the immune signalling profile that protects mosquitoes against *P. berghei* infection [9],[20].

### The Rel2-Short isoform controls *APL1A* gene expression and *P. falciparum* infection

The infection prevalence switch from non-permissive to permissive state for *P. falciparum* in the Rel2 knockdown mosquitoes might result from the removal of an unknown intrinsic molecular barrier to *P. falciparum* development that is frequent in wild-type *A. gambiae*. Because the knockdowns of *Rel2* and *APL1A* displayed the same phenotype, that is, elevated infection prevalence, we hypothesized that APL1A might be a mediator of the *P. falciparum* developmental blockade imposed by Rel2. Comparison of APL1A and Rel2 effects shows that mosquitoes silenced for each gene displayed comparable *P. falciparum* infection prevalences of 78% (Figure 2B) and 81% (Figure 5B) respectively, where the controls in both experiments were also comparable (57% and 59% prevalence, respectively). Thus, by magnitude of effect, the function of APL1A appears sufficient in itself to explain the protection by Rel2 against *P. falciparum*, suggesting that APL1A could be an important mediator of Rel2 protection against this parasite species.

To further explore the relationship between APL1A and Rel2 in immune signalling, we first examined transcriptional regulation of *APL1A*. There are two isoforms of Rel2 in *A. gambiae*: a Full-length form (Rel2-F) and a Short form (Rel2-S) lacking the inhibitory ankyrin repeats and death domain present in the full-length protein [21]. The *dsRel2* construct used above, which caused increased *P. falciparum* infection prevalence in treated mosquitoes (Figure 5B), targets a common region shared by both Rel2 isoforms. Gene silencing using the shared Rel2 construct also abolished transcript of *APL1A*, but not *APL1B* or *APL1C* (Figure 5C), indicating that transcript levels of paralog *APL1A* are under Rel2 control. To distinguish effects of the Rel2 isoforms we then used *dsRel2(Ank)*, a knockdown construct that targets the ankyrin domain present only in the full-length *Rel2-F* transcript, which consequently silences only *Rel2-F* but not the shorter *Rel2-S* [21]. Because *Rel2-F* includes the sequences present in *Rel2-S*, it is not possible to design a *Rel2-S* specific knockdown. In contrast to the absence of *APL1A* mRNA after treatment with *dsRel2*, mosquitoes treated with *dsRel2(Ank)* displayed similar *APL1A* transcript levels as *dsGFP* controls (Figure 5C). Corresponding to the ability to control *APL1A* expression, *dsRel2* treated mosquitoes displayed significantly increased *P. falciparum* infection prevalence while *dsRel2(Ank)* treated mosquitoes were not different from controls (Figure 5D). These results indicate that the Rel2-S and not Rel2-F isoform controls *P. falciparum* development in *A. gambiae*, and that the ability of a Rel2 isoform to control infection or not is correlated with its ability to regulate *APL1A* mRNA levels. There was no detectable effect of *dsAPL1A*, *dsAPL1B*, or *dsAPL1C* on *Rel2* transcript levels (data not shown), suggesting that *APL1A* is downstream of *Rel2-S* in an immune regulatory network that has unidirectional polarity. Taken together, these results suggest that some or most of the anti-*P. falciparum* activity of Rel2, and specifically of the Rel2-S isoform, is dependent upon APL1A function, and that the mechanism of anti-parasite protection probably operates by Rel2-S transcriptional control over *APL1A* expression.

## Discussion

Here we show that paralogs of a mosquito LRR gene family, *APL1*, display distinct activities against different species of the genus *Plasmodium*. Each *APL1* paralog is required for the full protection of mosquitoes against only one of the two malaria parasite lineages tested, and each *APL1* paralog protects by involvement in only one of two separate immune signalling pathways. Thus, paralog APL1A is required for protection against human *P. falciparum*, but not rodent *P. berghei* or *P. yoelii*, via the Imd/Rel2 pathway, while APL1C is required for protection against *P. berghei* and *P. yoelii* but not *P. falciparum*, and functions in the Toll/Rel1 pathway. These observations indicate that at least two reciprocal and mutually exclusive host-defense mechanisms, defined by the requirement for distinct *APL1* paralogs, protect mosquitoes from infection by different lineages of *Plasmodium* parasites.

It is recognized that invertebrate innate immunity distinguishes between broad groups of pathogens based on wide taxonomic and molecular differences, for example Gram-positive versus Gram-negative bacteria [16]. However, the current results demonstrate a previously unrecognized fine level of pathogen discrimination in the innate immune response of mosquitoes. We show that pathogens in the same genus, displaying high morphological and genetic relatedness, and with nearly identical mechanisms of cell invasion and development in the mosquito host, are nevertheless recognized and/or processed by mechanisms that require the function of closely related but distinct members of the *APL1* family. The *APL1* paralogs that are required for Rel1 or Rel2-dependent host protection, respectively, likely result from gene duplication and divergence [9]. The current study shows mutually exclusive protective function for two of the three known *APL1* paralogs, against three different pathogens in two evolutionary groups.

**Figure 6:**
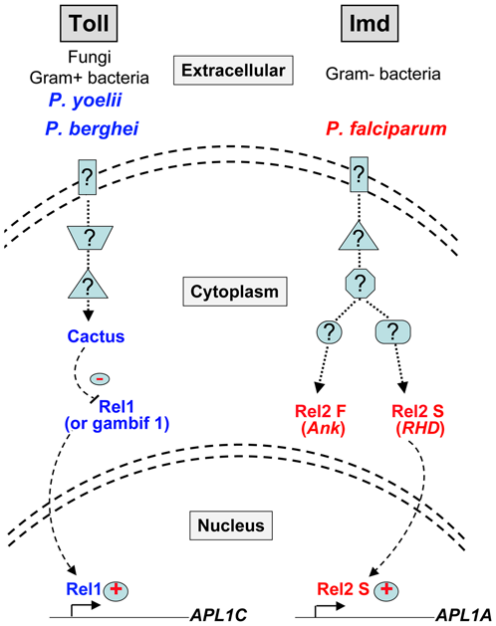
Model of APL1 paralog protection against pathogen classes in *A. gambiae*. In the cartoon, the rodent malaria parasites *P. berghei* and *P. yoelii*, which are experimental but not natural pathogens of *A. gambiae*, exemplify a class of pathogens whose development in the host is inhibited by an APL1C-dependent mechanism. The “C-like” class of pathogens against which APL1C protects should include other unknown natural *A. gambiae* pathogens. The APL1C pathway is depicted superimposed on the Toll pathway because the Cactus/Rel1 module regulates levels of *APL1C* transcript and protein [9], but the presumptive extracellular signals that elicit APL1C protection are unknown, as are the immune receptors and the signalling intermediates except Cactus/Rel1. *P. falciparum* protection is mediated also by unknown immune receptors and signalling intermediates, and is shown superimposed on the Imd pathway because part of the Rel2 signaling module is involved. Specifically, the short Rel2 isoform, Rel2-S, which contains the Rel-homology domain (RHD) is required for host protection against *P. falciparum* infection and for *APL1A* gene expression. In contrast, the long Rel2 isoform, Rel2-F, does not influence protection against *P. falciparum* or *APL1A* transcript levels, and thus is depicted as a functionally unrelated shunt from the APL1A-terminated pathway.

Overall, these data indicate that APL1A is required for full host protection against *P. falciparum*. This protection is dependent on Rel2-S but is independent of Rel2-F (Figure 6). Further work will be necessary to determine the structure of genetic and protein signaling in the Rel2-F subcircuit that regulates APL1-dependent host-protection, the mechanism of that protection, and the patterns of natural selection that shaped the *APL1* family. Recent work reported the requirement of APL1C for stability of a protein complex that includes the complement-related protein TEP1 and the LRR protein LRIM1 [23],[24]. The protein complex was tested only for protection against *P. berghei* and not *P. falciparum*, but in any case an APL1C-dependent complex could only be relevant for protection against the rodent parasite, because as shown here APL1C does not participate in protection against *P. falciparum*, and also because the other APL1C complex member, LRIM1, was previously shown to lack effect against *P. falciparum* [25]. Thus there is currently no reason to assume that APL1A-mediated protection against *P. falciparum* would be mechanistically similar to APL1C function in a soluble protein complex, particularly given their respective action in distinct immune signaling axes. We further found by functional analysis that APL2 (also known as LRRD7 [11]), another LRR-encoding candidate gene located within the PRI locus for which we found no protective effect against *P. berghei* [4], also had no significant effect upon *P. falciparum* development (data not shown).

These results do not imply that the functions of *APL1A* and *APL1C* are to protect exclusively against *P. falciparum* and the rodent malaria lineage, respectively, in a “gene-for-pathogen” system. Rather, it seems more likely that the function of the *APL1* paralogs illuminates different host-defense programs that act against repertoires of pathogens based on some shared characteristics, in other words, a “gene-for-pathogen-class” system. On this idea, APL1A protects not only against *P. falciparum* but also against as yet unknown “A-like” pathogens, and similarly for APL1C and “C-like” pathogens. We demonstrate here that activity of the correct APL1 factor is required to orient host defense towards human or rodent malaria parasites, respectively. There could be other gene families with similar fine-grained function against other pathogen classes, together yielding a potentially large combinatorial spectrum of protection profiles.

It is worth noting that APL1C protection against *P. berghei* and *P. yoelii* can not be the product of shared evolutionary history between the mosquito host and pathogen, because *A. gambiae* is not a natural vector of these parasites [26]. Thus, for *A. gambiae*, every experimental laboratory infection with rodent malaria represents exposure to a novel pathogen, and consequently the pathogen-resistance mechanisms in *A. gambiae* that are capable of protecting against rodent malaria species must have been shaped and maintained by interactions with other pathogens, and not by rodent malaria. Consequently, it would be interesting to know which natural pathogens of *Anopheles* are in the “C-like” group. In addition, does an APL1C ortholog protect the natural vector of *P. berghei*, *A. dureni* [26], against infection, and does it also function by a Rel1-dependent mechanism? In contrast, *A. gambiae* does have a shared evolutionary history with *P. falciparum*, and thus in principle host resistance mechanisms, including *APL1A*, could have been selected by that pathogen, although to date this has not been proven.

Further work will be required to distinguish between a variety of potential explanations for the reciprocal and mutually exclusive nature of *APL1* paralog protection displayed against different pathogens. For example, APL1 proteins could be soluble factors that recognize parasite surface molecular patterns. Their high level of structural polymorphism [9] could be consistent with tracking pathogen variation, but at least APL1C does not appear to detectably bind to the *P. berghei* surface [24] (and data not shown). Alternatively, APL1 proteins could be components of immune signaling nodes, where their activity against a given pathogen class might reflect transduction of upstream class-specific immune signals, or differential sensitivity to attack by pathogen-derived virulence factors. Here, the observed high rate of structural polymorphism might be driven by the need to escape inactivation by virulence factors, as hypothesized for the Relish cleavage complex in *Drosophila* [27],[28]. Further dissecting the theme of potential APL1 involvement in signaling rather than direct pathogen detection, APL1 gene products might function as guard proteins, in the terminology of plant host-defense studies [29], where the guard protein initiates an immune alarm if its binding partner is subjected to virulence factor attack. In this case, the factors guarded by APL1 proteins against virulence factor attack might be Rel1 and/or Rel2, which could be consistent with the requirement of APL1 activity for respective Rel function. Finally, it is possible that APL1 proteins might serve only as sensors of pathogen virulence factors, akin to the still-hypothetical plant Decoy Model [30]. On this model, the APL1 proteins would be under the functional constraints to i) serve as a molecular mimic of the actual virulence factor target in order to provoke a virulence factor attack, and ii) interact with a downstream host immune-signaling factor in order to transduce an immune signal in the event that the virulence factor is detected. In this case, the system of APL1 paralogs and their structural variants might be explained as the product of balancing selection to remain attractive for attack by an evolving virulence factor.

There is not yet a large enough dataset of pathogen-host interactions in mosquito, especially with natural pathogens like *P. falciparum*, to be able to distinguish between these or other possible models of APL1 protection. Indeed, to our knowledge this report represents the first time that even three pathogens have been used in a detailed functional study of mosquito host-defense. One current need is for repeated observations using distinct pathogens and different host genetic backgrounds. The phenotypic complexity of such studies requires better measures of phenotypic difference. The standard approach has been pooling of results from three replicate gene knockdown experiments to statistically test the effect of a given dsRNA, but simple pooling is not statistically valid if there are significant differences between replicates of the same treatment. Instead, here we first perform statistical comparison of replicates to determine whether pooling is appropriate, and if it is not (which can occur for many reasons), we use meta-analysis to compare treatment effects. Such an approach, combined with the use of a stringent significance cut-off (here we use p = 0.01), should increase comparability between experiments and between results from different laboratories.

The *APL1* genes were discovered as candidate factors within the PRI genetic locus that displays strong control over *P. falciparum* infection in nature [4]. In the current work we establish a validation trail that proceeds from that incriminated trait locus to the laboratory. We first functionally assayed *APL1* candidate gene protection in Cameroon against wild *P. falciparum*, then verified the protective phenotype using the same mosquito strain and dsRNA but with laboratory-cultured *P. falciparum* gametocytes. We were then able to functionally dissect some of the genes and signalling pathways underlying the original Cameroon result but under controlled laboratory conditions. We emphasize that the work presented herein does not prove that *APL1* underlies the phenotypic variation controlled by the PRI locus as detected in nature. For gene incrimination, it would be necessary at least to demonstrate a genetic association signal from the wild population. Thus, *APL1A* remains only a candidate gene, although strengthened as an interesting genetic candidate because of its functional properties as reported here. In contrast, based on their lack of functional effect, paralogs *APL1B* and *APL1C*, as well as the unrelated LRR protein APL2, can probably be excluded as genetic candidates for natural control of *P. falciparum* infection.

One interesting observation is that *APL1A* and *Rel2-S* knockdown phenotypes control *P. falciparum* infection prevalence at the usual infection levels that were obtained using either natural or *in vitro* parasites. This may be consistent with the previous speculation based on the genetic behaviour of the PRI locus in the wild population that mosquito resistance to *P. falciparum* could be the wild-type state, and that susceptibility might result from major loss-of-function mutations for an aspect of immune protection [4]. The binary infection prevalence switch observed here might have the features of such a gain- or loss-of-function system. It is also worth noting that, in terms of malaria transmission control, a mechanism that reduces infection prevalence should have more impact than reducing parasite numbers, because even one surviving oocyst is adequate for transmission.

## Methods

### Ethics statement

The use of human subjects was approved by the National Ethical Committee of Cameroon (protocol #039/CNE/MP/06). A signed individual written informed consent agreement was obtained from each subject or in the case of children from their parent or legally authorized representative before enrolment in the study.

### Mosquitoes

Mosquitoes were reared according to standard procedures [9]. The Ngousso strain was originally initiated with mosquitoes collected in Yaoundé, Cameroon in January 2006, and belongs to the M molecular and Forest chromosomal forms. The strain was reared in insectaries of OCEAC, Cameroon, or the CEPIA mosquito production facility, Institut Pasteur, France.

### Double-stranded RNA synthesis and injection

Double-stranded RNAs were synthesized from PCR amplicons using the T7 Megascript Kit (Ambion) as described previously [9]. The sequences of primers used for synthesis of dsRNA templates are in Supplementary Table S1. For each targeted gene, 200 ng of dsRNA (up to 207 nl volume, depending on the concentration) was injected into the thorax of cold-anesthetized 1–2 d-old *A. gambiae* females using a nano-injector (Nanoject II, Drummond Scientific). *dsGFP* or *dsLacZ* were used as controls. Injections were performed with a glass capillary needle as previously described [9],[31]. Mosquitoes were challenged with malaria parasites by feeding on the appropriate bloodmeal 4 d after dsRNA injection.

### Gene knockdown verification

The efficiency of the gene knockdown effect was monitored 4 d after dsRNA injection. cDNA synthesis was performed by using the M-MLV reverse transcriptase and random hexamers (Invitrogen). In each case, 500 ng of total RNA was used in triplicate assays. The triplicates were pooled and the mixture was used as template for PCR analysis. The sequences of primers used for gene knockdown verification are in Supplementary Table S1.

### Experimental infection of mosquitoes with wild *P. falciparum*

Gametocyte carriers were identified upon reading of thick blood smears from 5 to 11 year old children from the Mfou district, 30 km from Yaoundé, Cameroon. Carriers harboring over 20 gametocytes/µl were enrolled upon signature of an informed consent form by their legal guardian. Blood was collected by venipuncture in heparinized Vacutainer tubes, centrifuged at 2000 g for 5 minutes at 37°C, and the supernatant containing patient serum was replaced by non-immune AB serum. A blood volume of 350 µl was dispensed in glass feeders maintained at 37°C, and 5–6 d old female Ngousso strain mosquitoes were allowed to feed through a Parafilm membrane for 20 min. Only fully fed mosquitoes were retained for further analysis. Mosquitoes were maintained in an insectary at 27°C, 85% relative humidity on an 8% sucrose diet. The use of human subjects was approved by the National Ethical Committee of Cameroon.

### *P. falciparum* gametocyte culture and mosquito infection

*P. falciparum* isolate NF54 [32],[33] was cultured using the automated tipper-table system of Ponnudurai et al [34] as implemented in the CEPIA mosquito infection facility of Institut Pasteur. Briefly, a subculture of thawed NF54 stabilate was grown in 10 ml RPMI 1640 medium supplemented with 25 mM HEPES and L-glutamine, 10% heat-inactivated human serum, and sodium bicarbonate at 0.2% final concentration under a constant gas regime (5% CO2, 1% O2, 94% N2). Fresh anonymous erythrocytes obtained from blood banks were added to 7% final concentration. Fourteen days after initiating the subculture, gametocyte maturity was tested by exflagellation of microgametes, and parasitemia and numbers of mature male and female gametocytes were counted on Giemsa stained slides.

Ten ml of culture was centrifuged at 2000 rpm, and the cell pellet was resuspended in an equal volume of normal type AB human serum. The infected erythrocytes were added to fresh erythrocytes in AB human serum and were transferred to a membrane feeder warmed to 37°C. Mosquitoes were allowed to feed for 15 minutes, and only fully engorged females were used for further analysis. Bloodfed mosquitoes were maintained on 10% sucrose solution supplemented with 0.05% para-amino benzoic acid.

### Rodent malaria infection

Mosquitoes were fed on mice infected with *P. berghei* strain PbGFPCON [35], which constitutively expresses green fluorescent protein (GFP), or with *P. yoelii yoelii* strain 17XNL at 8–12% parasitemia with mature gametocytes. Mosquitoes were maintained at 21°C (*P. berghei*) or 24°C (*P. yoelii*) and 70% relative humidity on 10% sucrose supplemented with 0.05% para-amino benzoic acid.

### Analysis of phenotypes

Midguts were dissected at 7–8 d post-infection, and for *P. falciparum* or *P. yoelii*: stained in 0.4% mercurochrome and the number of oocysts counted by light microscopy; or for *P. berghei*: no mercurochrome and counting by fluorescence microscopy. A mosquito was considered infected if it harbored at least one oocyst. Prevalence of infection is defined as the proportion of infected mosquitoes among the total dissected mosquitoes. Infection intensity is defined as the number of oocysts per mosquito, determined using mosquitoes harboring at least one oocyst. It is necessary to exclude the uninfected fraction of mosquitoes from analysis of intensity, because otherwise the result confounds infection prevalence and parasite intensity effects.

For each gene-silencing experiment at least 30 surviving mosquitoes were counted for oocyst load, and at least three independent replicate experiments were performed per experimental treatment. For statistical analysis, first all experiments were tested for statistical differences within treatments across replicates. If no differences between replicates were detected, replicates were pooled and differences in prevalence were tested using Chi Square and differences in intensity were tested using non-parametric Wilcoxon Mann-Whitney or Kruskal Wallis ANOVA on Ranks. If differences between replicates were detected, which can occur due to the inherent variability of *Plasmodium* experimental infections, replicates were analyzed independently using the tests described above and p values from independent tests of significance were combined using the meta-analytical approach of Fisher [36], and this combined p value is reported here. By either approach, the threshold for significance was defined as p = 0.01.

## Supporting Information

Table S1 Primers used for synthesis of double-stranded RNAs (prefix T7, T7 portion underlined) and for knockdown verification of target genes (suffix V). Final suffix indicates forward, F, or reverse, R, sense of primers. Where no V primers are listed (Rel1, Rel2, Rel2(Ank), and Cactus), the synthesis primers without T7 sequences were also used for verification.
(0.04 MB DOC)

Table S2 Infection prevalence, measured as the fraction of mosquitoes with at least one midgut oocyst, analyzed using Chi Square. Oocyst intensity is analyzed only in mosquitoes with ≥1midgut oocyst and is analyzed using non-parametric Wilcoxon Mann Whitney (WMW) tests. Analyses are presented by the figure number in which the data appears. Values lower than the significance threshold of p = 0.01 are shown in bold. 1: p values from pooling of replicate experiments for statistical analysis where there were not significant differences between replicates within treatments. 2: p values from independent analyses of replicate experiments and combination of independent p values using the meta-analysis method of Fisher (see Methods). This approach was used when there were significant differences within treatment across replicates.
(0.05 MB DOC)

Table S3 Descriptive results of infections used in corresponding figures as indicated. Data are shown from pooled replicate infections. Median and range shown are for mosquitoes with at least one oocyst. Proportions of infected mosquitoes (infection prevalence) are shown in the figures.
(0.03 MB DOC)

Figure S1 Constitutive activation of Rel1 by silencing of Cactus does not influence *P. falciparum* development. A) *dsCactus* treatment efficiently silences cactus gene expression. B) The Cactus-depleted state, previously shown to constitutively activate Rel1 (see Results) does not affect the efficiency of *P. falciparum* development in *A. gambiae*.
(3.46 MB TIF)
