# Supplementary material for: Fine Pathogen Discrimination within the APL1 Gene Family Protects Anopheles gambiae against Human and Rodent Malaria Species
Source: PLoS Pathog. 2009 Sep 11;5(9):e1000576. doi: 10.1371/journal.ppat.1000576 (PMC2734057; doi:10.1371/journal.ppat.1000576)
Supplement: Table S1 — Primers used for synthesis of double-stranded RNAs (prefix T7, T7 portion underlined) and for knockdown verification of target genes (suffix V). Final suffix indicates forward, F, or reverse, R, sense of primers. Where no V primers are listed (Rel1, Rel2, Rel2(Ank), and Cactus), the synthesis primers without T7 sequences were also used for verification. (0.04 MB DOC) [file ppat.1000576.s001.doc]

| T7-GFP-F | GAATTGTAATACGACTCACTATAGGGCATGGTGAGCAAGGGCGAG |
| --- | --- |
| T7-GFP-R | GAATTGTAATACGACTCACTATAGGGCTTACTTGTACAGCTCGTC |
| T7-wAPL1-F | TAATACGACTCACTATAGGATATAACACTAAACAACC |
| T7-wAPL1-R | TAATACGACTCACTATAGGAAAGATCCACGTCAACCA |
| T7-APL1A-F | TAATACGACTCACTATAGGACTACCACCAGCCGAAAGATG |
| T7-APL1A-R | TAATACGACTCACTATAGGATCTGGTCTTGTATAGTACAATGG |
| T7-APL1B-F | TAATACGACTCACTATAGGACTCGCAAAGCTCAGCAAACAC |
| T7-APL1B-R | TAATACGACTCACTATAGGAGTGAGAACAAATAAGTTCAAAGTCC |
| T7-APL1C-F | TAATACGACTCACTATAGGAGGCCAAGAAGAACCGCAATCC |
| T7-APL1C-R | TAATACGACTCACTATAGGATCACAGTGATTTCAGGGTGTGC |
| S7’A | AGGCGATCATCATCTACGTGC |
| S7 B | GTAGCTGCTGCAAACTTCGG |
| APL1A-VF | GTAAACGAGCTGAGGACTGCGGTGCAGC |
| APL1A-VR | TCTGGTCTTGTATAGTACAATGGAACC |
| APL1B-VF | ACTCGCAAAGCTCAGCAAACAC |
| APL1B-VR | GTGAGAACAAATAAGTTCAAAGTCC |
| APL1C-VF | CTGCTGCAGGGGCTACACGCC |
| APL1C-VR | GGCCCAAGTAACATCATACAC |
| T7-Rel1-F | TAATACGACTCACTATAGGGCAACAGAACCCGTTCAACTTGC |
| T7-Rel1-R | TAATACGACTCACTATAGGGAATGGATGCTTACGGGCTAACG |
| T7-Rel2-F | TAATACGACTCACTATAGGGCAACAGCAGCAACAACATC |
| T7-Rel2-R | TAATACGACTCACTATAGGGCACAGGCACACCTGATTGA |
| T7-Rel2(Ank)-F | TAATACGACTCACTATAGGAATCCGACGCAACGATACG |
| T7-Rel2(Ank)-R | TAATACGACTCACTATAGGGACCGCAATGTGAAGGATG |
| T7-Cactus-F | TAATACGACTCACTATAGGTGGTGCGTCGATTGCTGG |
| T7-Cactus-R | TAATACGACTCACTATAGGCTTTCGTTCAAGTTCTGTGC |

**Supplementary Table S1.** Primers used for synthesis of double-stranded RNAs (prefix T7, T7 portion underlined) and for knockdown verification of target genes (suffix V). Final suffix indicates forward, F, or reverse, R, sense of primers. Where no V primers are listed (Rel1, Rel2, Rel2(Ank), and Cactus), the synthesis primers without T7 sequences were also used for verification.
