# Supplementary material for: Fine Pathogen Discrimination within the APL1 Gene Family Protects Anopheles gambiae against Human and Rodent Malaria Species
Source: PLoS Pathog. 2009 Sep 11;5(9):e1000576. doi: 10.1371/journal.ppat.1000576 (PMC2734057; doi:10.1371/journal.ppat.1000576)
Supplement: Table S2 — Infection prevalence, measured as the fraction of mosquitoes with at least one midgut oocyst, analyzed using Chi Square. Oocyst intensity is analyzed only in mosquitoes with ≥1midgut oocyst and is analyzed using non-parametric Wilcoxon Mann Whitney (WMW) tests. Analyses are presented by the figure number in which the data appears. Values lower than the significance threshold of p = 0.01 are shown in bold. 1: p values from pooling of replicate experiments for statistical analysis where there were not significant differences between replicates within treatments. 2: p values from independent analyses of replicate experiments and combination of independent p values using the meta-analysis method of Fisher (see Methods). This approach was used when there were significant differences within treatment across replicates. (0.05 MB DOC) [file ppat.1000576.s002.doc]

|  | *p* values | |  | |
| --- | --- | --- | --- | --- |
|  | Infection Prevalence | Oocyst Intensity | |  |
| Figure 1 |  |  | |  |
| *LacZ*kd vs *APL1*kd | **0.006**^2^ | 0.926^2^ | |  |
|  |  |  | |  |
| Figure 2 |  |  | |  |
| *GFP*kd vs *APL1A*kd | **0.001**^1^ | 0.694^2^ | |  |
| *GFP*kd vs *APL1B*kd | 0.755^1^ | 0.850^2^ | |  |
| *GFP*kd vs *APL1C*kd | 0.704^1^ | 0.331^2^ | |  |
|  |  |  | |  |
| Figure 3 |  |  | |  |
| *GFP*kd vs *APL1A*kd | **0.0004**^2^ | 0.049^2^ | |  |
| *GFP*kd vs *wAPL1*kd | **0.0007**^2^ | 0.012^2^ | |  |
| *wAPL1*kd vs *APL1A*kd | 0.883^2^ | 0.793^2^ | |  |
|  |  |  | |  |
| Figure 4 |  |  | |  |
| *GFP*kd vs *APL1A*kd | 0.188^2^ | 0.107^2^ | |  |
| *GFP*kd vs *APL1B*kd | 0.025^2^ | 0.627^2^ | |  |
| *GFP*kd vs *APL1C*kd | **2.825e-07** ^2^ | **0.0004^2^** | |  |
|  |  |  | |  |
| Figure 5 |  |  | |  |
| *GFP*kd vs *Rel1*kd | 0.908^1^ | 0.127^2^ | |  |
| *GFP*kd vs *Rel2*kd | **0.0002**^2^ | 0.259^2^ | |  |
| *GFP*kd vs *Rel2(Ank)*kd | 0.805^2^ | 0.301^2^ | |  |
|  |  |  | |  |
|  |  |  | |  |

**Supplementary Table S2.** Infection prevalence, measured as the fraction of mosquitoes with at least one midgut oocyst, analyzed using Chi Square. Oocyst intensity is analyzed only in mosquitoes with ≥ 1midgut oocyst and is analyzed using non-parametric Wilcoxon Mann Whitney (WMW) tests. Analyses are presented by the figure number in which the data appears. Values lower than the significance threshold of p=0.01 are shown in bold.

1. *p* values from pooling of replicate experiments for statistical analysis where there were not significant differences between replicates within treatments.
2. *p* values from independent analyses of replicate experiments and combination of independent *p* values using the meta-analysis method of Fisher (see Methods). This approach was used when there were significant differences within treatment across replicates.
