# Supplementary material for: Fine Pathogen Discrimination within the APL1 Gene Family Protects Anopheles gambiae against Human and Rodent Malaria Species
Source: PLoS Pathog. 2009 Sep 11;5(9):e1000576. doi: 10.1371/journal.ppat.1000576 (PMC2734057; doi:10.1371/journal.ppat.1000576)
Supplement: Table S3 — Descriptive results of infections used in corresponding figures as indicated. Data are shown from pooled replicate infections. Median and range shown are for mosquitoes with at least one oocyst. Proportions of infected mosquitoes (infection prevalence) are shown in the figures. (0.03 MB DOC) [file ppat.1000576.s003.doc]

Median oocysts/infected mosquito Range of oocysts/infected mosquito

*Lacz*kd 6 1-53

**Figure 1**

*wAPL1*kd 7 1-63

*GFP*kd 2 1-80

*APL1A*kd 2 1-36

**Figure 2**

*APL1B*kd 2 1-55

*APL1C*kd 1 1-34

*GFP*kd 4 1-100

**Figure 3**

*APL1A*kd 3 1-106

*wAPL1*kd 3 1-116

*GFP*kd 2 1-10

**Figure 4**

*APL1A*kd 2.5 1-12

*APL1B*kd 1 1-15

*APL1C*kd 9 1-300

*GFP*kd 2 1-70

*Rel1*kd 2 1-24

**Figure 5**

*Rel2*kd 3 1-58

*Rel2(Ank)*kd 1.5 1-30

*GFP*kd 2.5 1-93

**S1**

*Cactus*kd 2 1-46

**Supplementary Table S3.** Descriptive results of infections used in corresponding figures as indicated. Data are shown from pooled replicate infections. Median and range shown are for mosquitoes with at least one oocyst. Proportions of infected mosquitoes (infection prevalence) are shown in the figures.
