# Supplementary figures and images for: Fine Pathogen Discrimination within the APL1 Gene Family Protects Anopheles gambiae against Human and Rodent Malaria Species
Source: PLoS Pathog. 2009 Sep 11;5(9):e1000576. doi: 10.1371/journal.ppat.1000576 (PMC2734057; doi:10.1371/journal.ppat.1000576)

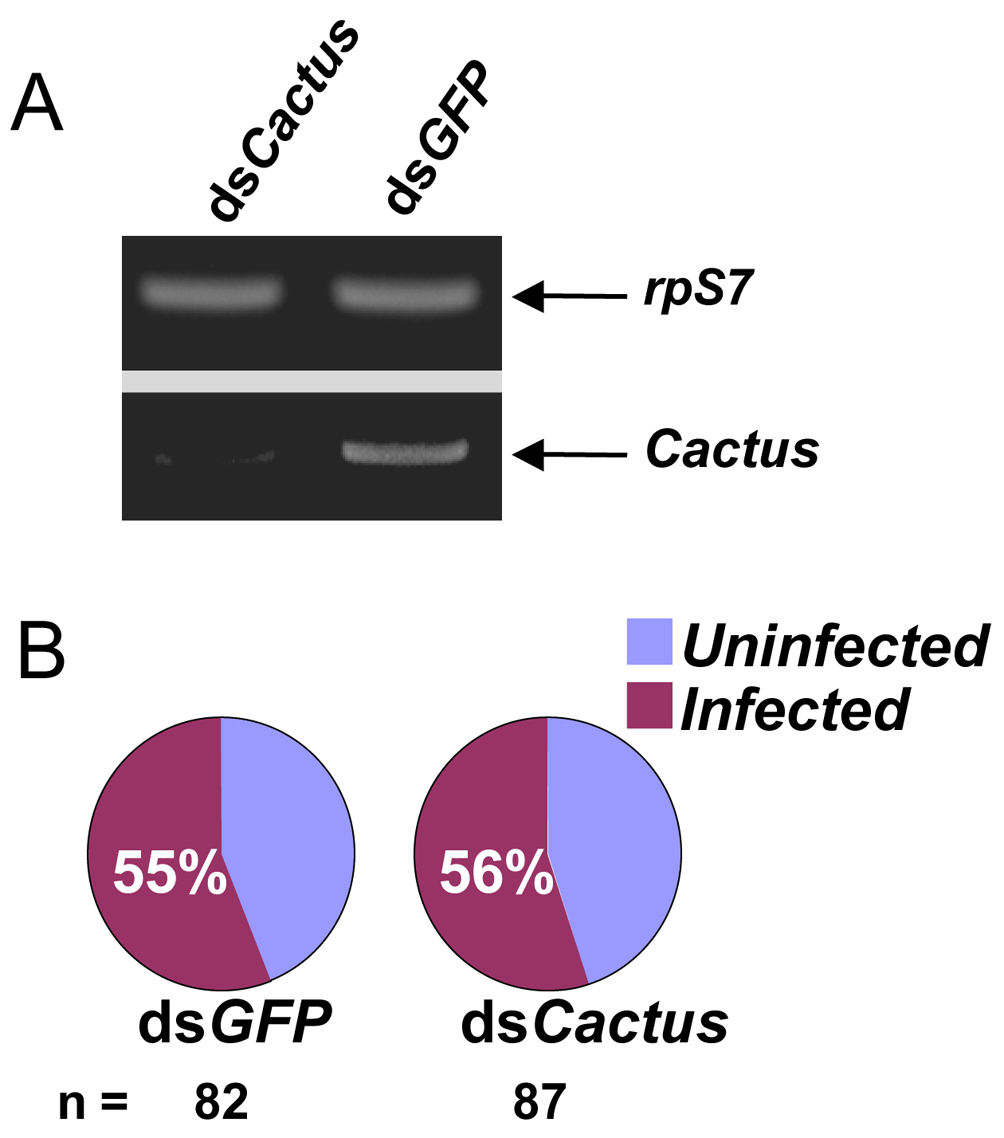

Supplement: Figure S1 — Constitutive activation of Rel1 by silencing of Cactus does not influence P. falciparum development. A) dsCactus treatment efficiently silences cactus gene expression. B) The Cactus-depleted state, previously shown to constitutively activate Rel1 (see Results) does not affect the efficiency of P. falciparum development in A. gambiae. (3.46 MB TIF) [file ppat.1000576.s004.tif]
